# Highly Efficient CoFeP Nanoparticle Catalysts for Superior Oxygen Evolution Reaction Performance

**DOI:** 10.3390/nano14171384

**Published:** 2024-08-24

**Authors:** Abhishek Meena, Abu Talha Aqueel Ahmed, Aditya Narayan Singh, Vijaya Gopalan Sree, Hyunsik Im, Sangeun Cho

**Affiliations:** 1 Division of System Semiconductor, College of AI Convergence, Dongguk University, Seoul 04620, Republic of Korea; 2 Department of Energy and Materials Engineering, Dongguk University, Seoul 04620, Republic of Korea; 3 Department of Physics, Dongguk University, Seoul 04620, Republic of Korea

**Keywords:** oxygen evolution reaction, non-precious metal catalyst, phosphorization, amorphous/crystalline composite

## Abstract

Developing effective and long-lasting electrocatalysts for oxygen evolution reaction (OER) is critical for increasing sustainable hydrogen production. This paper describes the production and characterization of CoFeP nanoparticles (CFP NPs) as high-performance electrocatalysts for OER. The CFP NPs were produced using a simple hydrothermal technique followed by phosphorization, yielding an amorphous/crystalline composite structure with improved electrochemical characteristics. Our results reveal that CFP NPs have a surprisingly low overpotential of 284 mV at a current density of 100 mA cm^−2^, greatly exceeding the precursor CoFe oxide/hydroxide (CFO NPs) and the commercial RuO_2_ catalyst. Furthermore, CFP NPs demonstrate exceptional stability, retaining a constant performance after 70 h of continuous operation. Post-OER characterization analysis revealed transformations in the catalyst, including the formation of cobalt–iron oxides/oxyhydroxides. Despite these changes, CFP NPs showed superior long-term stability compared to native metal oxides/oxyhydroxides, likely due to enhanced surface roughness and increased active sites. This study proposes a viable strategy for designing low-cost, non-precious metal-based OER catalysts, which will help advance sustainable energy technology.

## 1. Introduction

The pressing challenges of environmental pollution and the depletion of traditional energy sources have intensified the research and development of green energy technologies [1]. Among these, hydrogen as a green energy source has appeared as a compelling alternative due to its abundant availability, high energy density, and zero-emission usage. Sustainable hydrogen production through water splitting is widely seen as a favorable approach for curbing the energy crisis while combating environmental degradation [2]. As global energy systems transition to low-carbon solutions, hydrogen stands out as a green and efficient energy source that can replace fossil fuels [2,3].

An environmentally friendly method for producing hydrogen is provided by electrochemical water splitting, which consists of the two essential half-cell reactions known as the hydrogen evolution reaction (HER) and oxygen evolution reaction (OER) [4,5]. Nevertheless, the efficiency of the water-splitting process is significantly compromised by the slow kinetics associated with the OER. The development of high-efficiency OER electrocatalysts using non-precious metals is urgently needed to improve performance [6]. Due to their exceptional performance, noble metals such as RuO_2_ and IrO_2_ have been known as commercial catalysts. However, the high cost and scarcity of these noble materials pose significant obstacles to their large-scale commercialization [4,7].

Given these difficulties, non-precious metal catalysts are gaining popularity [8]. In particular, non-noble transition metal phosphides (TMPs) hold great potential as substitutes for noble metal-based electrocatalysts for OER with excellent OER activity and stability [9]. The remarkable electrocatalytic activity, affordability, and abundance of TMPs have attracted great interest. The metal sites and anions on the TMP surface act as specific hydride and proton receptors and contribute to the high activity [10,11,12,13,14]. Phosphorus is one of the most abundant nonmetallic elements and has several benefits because it is inexpensive and widely available [15,16]. When it comes to water splitting, phosphorus atoms can draw electrons away from the metal surface. This makes it easier for protons and oxygen-containing intermediates to be adsorbed, which increases catalytic activity [17,18]. Despite significant advancements, many previous TMP catalysts have exhibited poor activity and stability in OER. This challenge underscores the ongoing need for an improved catalyst design. To address these issues, various strategies have been explored to enhance the overall efficiency of TMPs for OER, including optimizing their electronic structure, modifying surface properties, and developing hybrid materials to improve both catalytic activity and stability.

According to recent research, bimetallic or mixed-metal phosphides have better catalytic qualities than their monometallic counterparts because of advantageous synergistic effects [19,20]. In this context, low-overpotential electrocatalysts for the OER have been reported to include cobalt, iron, and their corresponding bimetallic phosphides [21]. Bimetallic transition metals introduce unique chemistry to the OER. For example, the addition of Fe to Ni-based electrocatalysts significantly changed the OER performance and increased the film conductivity by a factor of thirty in one study [22].

Moreover, it is well known that new nanostructures can offer notable structural benefits that greatly improve catalytic performance [23,24]. Catalysts with a larger density of accessible active sites must be engineered to generate highly effective OER activity [25,26].

This can be achieved using creative design techniques that enhance surface area and expose more catalytic sites [27]. Enhancing mass transfer and charge transfer capacities is also essential since it enables the effective delivery of reactants and electrons to the active sites, speeding up the kinetics of the reaction [28,29]. A singular interface that promotes synergistic interactions and increases catalytic activity and stability can be produced by combining both crystalline and amorphous phases in a single nanostructure [15,29]. While amorphous areas might offer flexible environments that adapt to the dynamic conditions of the reaction, crystalline phases can offer well-defined paths for electron transport [30,31,32,33]. These cutting-edge nanostructure designs not only elevate the catalyst’s intrinsic catalytic properties but also significantly enhance its long-term durability, particularly under high-current-density conditions. Such advancements are pivotal in the development of next-generation electrocatalysts, which are essential for addressing the complex challenges associated with sustainable energy conversion and storage technologies. As this field of study continues to evolve, the exploration and refinement of innovative nanostructures with tailored characteristics will remain a key focus, driving the pursuit of even higher catalytic performance.

In this work, novel CFP NPs prepared from CFO NPs are presented as excellent catalysts for the OER. First, a simple hydrothermal technique was used to fabricate CFO nanosheets. The solid composite nanosheets were then converted into CFP NPs via phosphorization. The resulting nanostructured CFP NP catalyst exhibits high OER activity due to its increased electrochemical surface area and abundance of exposed active sites. During the OER, iron and cobalt serve as active centers. Cobalt’s electron donation to Fe optimizes the electronic structure for improved performance. Furthermore, we hypothesize that this increased electrocatalytic activity is mostly due to the amorphous and crystalline phases that emerge in a single CFP NP structure when phosphorization occurs at a low temperature. Therefore, CFP NPs exhibit excellent catalytic activity, making them a viable option for the sustainable production of hydrogen. The resulting OER electrocatalyst outperforms the commercial RuO_2_ catalyst in terms of OER activity and exhibits an exceptionally low overpotential of 284 mV at a current density of 100 mA cm^−2^. Additionally, this catalyst exhibits remarkable durability, operating continuously for over 70 h at a high current density of 100 mA cm^−2^.

## 2. Materials and Methods

### 2.1. Materials and Reagents

No additional purification was required. All analytical grade chemicals were used exactly as supplied. The following reagents were purchased from Sigma Aldrich (Seoul, Republic of Korea): potassium hydroxide, sodium hydroxide, iron(III) chloride, ruthenium oxide powder (RuO_2_), cobalt(II) chloride, sodium hydroxide, 5 wt% Nafion, and sodium hypophosphite monohydrate. All aqueous solutions were prepared with double-deionized water (DDW). As-delivered nickel foam from MTI Corporation (Seoul, Republic of Korea) was used.

### 2.2. Material Characterization

X-ray diffraction (XRD) was used to examine the structural and crystallographic characteristics of the catalytic electrode films using a Rigaku Smartlab device (Tokyo, Japan). CuKα radiation with a wavelength (λ) of 1.54056 A° was used to gather XRD spectra, which were scanned at a rate of 2° min^−1^ throughout a 2θ angle range of 10° to 80°. The voltage and current used by the instrument were 40 kV and 30 mA, respectively. The morphology and composition of the materials were investigated using energy-dispersive X-ray spectroscopy (EDS) and field emission scanning electron microscopy (FESEM) using a Hitachi High-Technologies (SU-8220) microscope (Tokyo, Japan). At a low magnification of ×5000, EDS spectra were collected while the instrument was operating at 15 kV. A JEM-2100 F (Tokyo, Japan) microscope from JEOL was used to capture high-resolution transmission electron microscopy (HRTEM) pictures while operating at an acceleration voltage of 200 kV. The oxidation states of the elements were ascertained by X-ray photoelectron spectroscopy (XPS), with binding energies being compared to the carbon contamination peak (C 1s at 284.33 eV). A Thermo Fisher (Waltham, MA, USA) (K-alpha) instrument was used to perform the XPS measurements.

### 2.3. Synthesis of CFO NPs

A straightforward hydrothermal technique was used to create cobalt–iron oxide/hydroxide nanoparticles (CFO NPs). To ensure a homogenous mixture, iron (III) chloride (0.11 M) and cobalt(II) chloride (0.22 M) were first dissolved in 100 mL of deionized water and vigorously stirred for 15 min. The mixture was then continuously stirred for an hour while a 2 M NaOH solution, dissolved in 50 mL of deionized water, was added dropwise to create a CoFe suspension. To eliminate any contaminants, distilled water, and ethanol were used to wash the resultant suspension. The CoFe suspension was then quickly added to 180 milliliters of deionized water and an aqueous solution of NaOH (2.5 M). Three 100 mL Teflon-lined stainless-steel autoclaves were filled with this solution, sealed, and kept at 160 °C for a full day. The CFO nanoparticles were collected once they had cooled to room temperature, thoroughly cleaned with ethanol and water, and then dried for ten hours at 60 °C.

### 2.4. Synthesis of CFP NPs

The phosphorization of the CFO NP precursor enabled the synthesis of the CFP NPs. To be precise, 50 mg of CFO nanoparticles and 500 mg of NaPO_2_H_2_·H_2_O were placed in a porcelain boat at a distance of 3–4 cm, with the CFO NPs placed downstream and the NaPO_2_H_2_·H_2_O upstream in a tube furnace. At a ramp rate of 3 °C per minute, the system was then heated to 350 °C for 2 h while exposed to an argon (Ar) atmosphere to prepare the CFP NPs.

### 2.5. Electrochemical Measurements

For electrochemical studies, a three-electrode system operated in a 1 M KOH solution was used. The reference electrode was a Hg/HgO electrode, and the counter electrode was a graphite rod. The working electrode consisted of nickel foam (NF), onto which the synthesized powder catalysts were directly loaded. With an iR compensation of 95 percent, linear sweep voltammetry (LSV) was performed with a scan rate of 2 mV s^−1^. Chronopotentiometry was used to assess the long-term stability of the catalysts at a constant current density of 100 mA cm^−2^. The potentials were converted to the reversible hydrogen electrode (RHE) scale by measuring them against the Hg/HgO electrode using the following equation (Figure S1): E(RHE) = E(Hg/HgO) + 0.904.

Furthermore, electrochemical impedance spectroscopy (EIS) was performed in a 1 M KOH solution at a potential of 1.53 V, with an AC voltage amplitude of 10 mV over a frequency range of 10 kHz to 10 MHz. The NF was carefully cleaned by submerging it in a 1 M hydrochloric acid (HCl) solution for 40 min to eliminate impurities, hydroxides, and surface oxides before performing all electrochemical tests. After that, to make sure that all traces of acid were completely removed, the NF was thoroughly rinsed with ethanol and deionized water. To ensure a flawless surface, the foam was finally dried in a vacuum for 6 h.

## 3. Results and Discussion

### 3.1. Crystallography and Morphology

**Figure 1:**
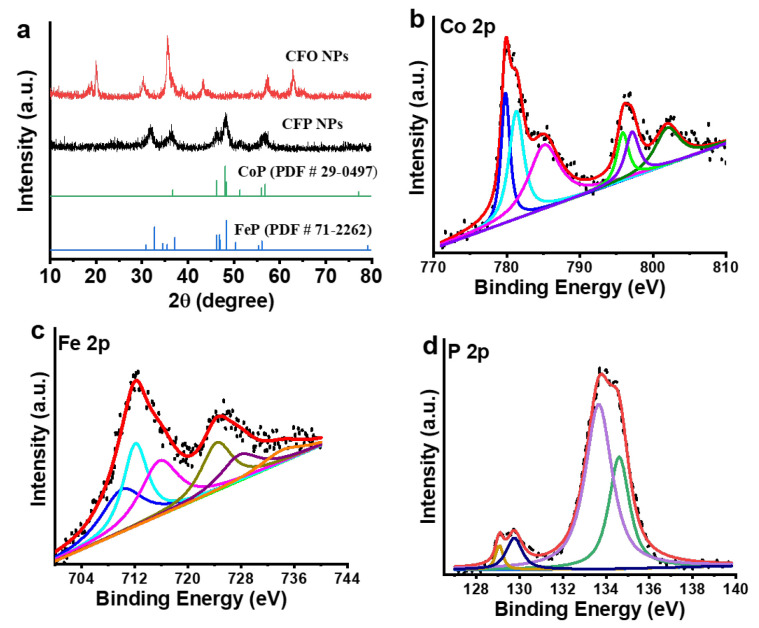
(**a**) XRD patterns of as-prepared samples of CFO and CFP NPs. (**b**–**d**) High-resolution XPS spectra for constituent elements: (**b**) Co 2p, (**c**) Fe 2p, and (**d**) P 2p of CFP NPs.

The crystalline structures of CFO and CFP NPs were investigated using X-ray diffraction (XRD). Figure 1a shows the XRD patterns for both CFO and CFP NPs. The CFO nanoparticles have discrete diffraction peaks that correspond to cobalt–iron oxide/hydroxide phases, proving their identification prior to phosphorization [34,35,36]. After treatment with NaH_2_PO_2_·H_2_O, CFO nanoparticles successfully transition into CFP nanoparticles, as evidenced by the changes in the XRD patterns. The XRD pattern of CFP NPs shows significant peaks at 31.8°, 36.3°, 48.1°, and 56.5°, all of which correlate to CoP (PDF # 29-0497), indicating the presence of cobalt phosphide. Additionally, the peaks at 32.9°, 37.2°, and 48.3° correspond to FeP (PDF # 71-2262), confirming the synthesis of iron phosphide. The emergence of these specific diffraction peaks suggests that the CFO NPs were successfully phosphorized, resulting in the creation of CoP and FeP phases in the CFP NPs [37,38,39]. This change not only confirms the synthesis of the target bimetallic phosphides but also demonstrates the efficiency of the phosphorization process in changing the crystallographic structure of the precursor material.

To further investigate the electronic states and elemental composition of the CFP NPs, X-ray photoelectron spectroscopy (XPS) was used, offering precise insights into the surface chemistry and bonding environment of the constituent elements. The XPS spectra for Co 2p (Figure 1b) show strong peaks at binding energies (BEs) of 779.9 eV for Co 2p_3/2_ and 795.8 eV for Co 2p_1/2_, which are typical of Co-P bonds and indicate good cobalt integration into the phosphide matrix. Additionally, the appearance of peaks at 781.3 eV for Co 2p_3/2_ and 797.1 eV for Co 2p_1/2_ suggests the presence of oxidized cobalt species on the electrocatalyst’s surface, likely formed due to surface oxidation when exposed to air. Satellite peaks at 785.3 eV and 802.0 eV are also observed, confirming the complex surface chemistry of the CFP NPs [40]. Similarly, the Fe 2p XPS spectrum (Figure 1c) provides crucial insights into the electronic state of iron within CFP NPs. The Fe 2p spectrum exhibits peaks at 710.0 eV and 724.3 eV, corresponding to Fe 2p_3/2_ and Fe 2p_1/2_ of Fe^2+^, respectively. Additionally, the peaks at 712.1 eV and 727.7 eV are attributed to Fe 2p_3/2_ and Fe 2p_1/2_ of Fe^3+^, indicating the presence of oxidized iron species. Two additional peaks at 715.7 eV and 734.2 eV can be assigned to the satellite peaks, which are characteristic of Fe^3+^ species. These satellite peaks confirm the presence of multiple oxidation states of iron in CFP NPs [40,41]. The P 2p XPS spectrum (Figure 1d) further confirms the successful phosphorization process. The high-resolution P 2p spectrum of CFP NPs reveals four distinct peaks after deconvolution. The peaks at 129.03 eV and 129.78 eV correspond to P 2p_3/2_ and P 2p_1/2_, respectively, while the peaks at 133.3 eV and 134.1 eV are attributed to P–O bonds, which likely result from the oxidation of CFP NPs [42,43,44].

**Figure 2:**
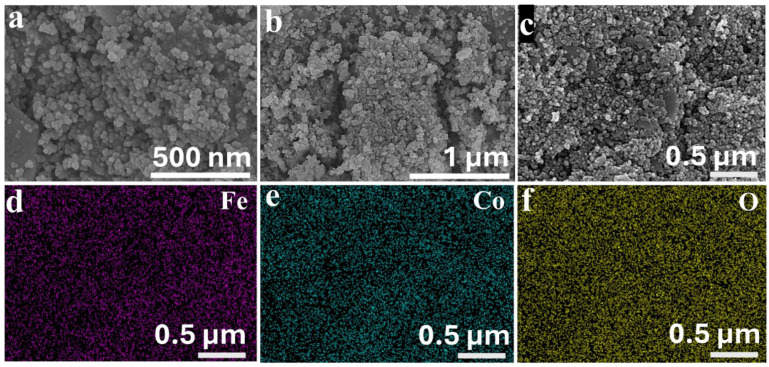
(**a**,**b**) FESEM images of CFO NPs. EDS elemental mapping of the CFO NPs: (**c**) SEM image and (**d**–**f**) corresponding EDS-based elemental mapping of Fe, Co, and O, respectively.

**Figure 3:**
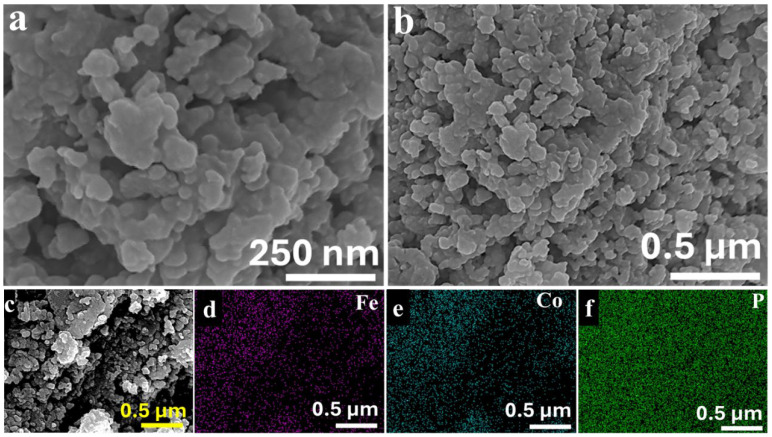
(**a**,**b**) FESEM images of CFP NPs. EDS elemental mapping of the CFP NPs: (**c**) SEM image and (**d**–**f**) corresponding EDS-based elemental mapping of Fe, Co, and P, respectively.

The morphological properties of the CFO and CFP NPs were rigorously investigated using scanning electron microscopy (SEM) and transmission electron microscopy (TEM) to acquire insight into the structural modifications caused by the phosphorization process. SEM images of CFO NPs (Figure 2a–c) show a well-defined, consistent shape with the development of distinct nanoparticles. Uniformity is important because it ensures consistent physical and chemical qualities throughout the sample. Complementary energy-dispersive X-ray spectrometry (EDS) mapping (Figure 2d–f and Figure S2) supports the homogenous distribution of Fe, Co, and O within the CFO NPs, demonstrating a successful synthesis of the cobalt–iron oxide/hydroxide phase. Significant changes in morphology were found following phosphorization. The SEM pictures of the CFP NPs (Figure 3a,b) reveal a complete disruption of the previously homogeneous structure. The nanoparticles have an irregular and less defined shape, as is typical of materials undergoing phase transformations such as the transition from oxide to phosphide. This morphological shift indicates that the phosphorization process has a considerable impact on the physical structure of the CFO NPs in addition to altering their chemical composition. The EDS mapping of the CFP NPs (Figure 3c–f and Figure S3) gives additional evidence of effective phosphorization. The elemental maps reveal a uniform and even distribution of cobalt (Co), iron (Fe), and phosphorus (P) across the nanoparticles, indicating that the phosphorization process successfully integrated phosphorus into the material.

**Figure 4:**
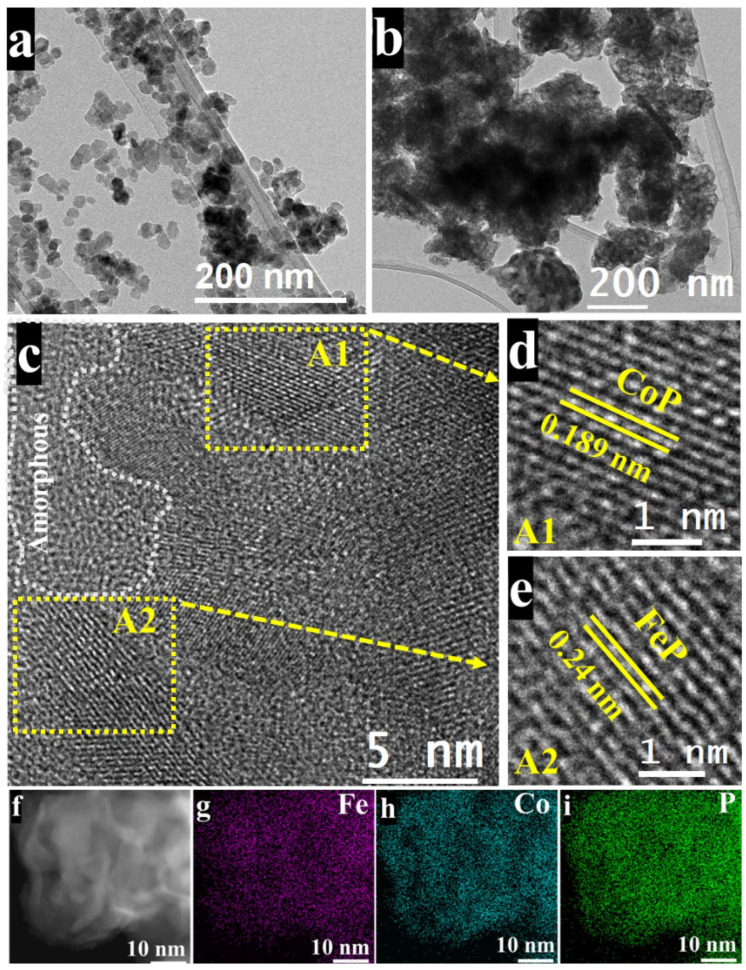
(**a**) HRTEM image of CFO NPs. (**b**) HRTEM image of CFP NPs. (**c**) High-resolution TEM image of CFP NPs. (**d**) Zoomed-in HRTEM image highlighting region A1, corresponding to the CoP phase. (**e**) Zoomed-in HRTEM image highlighting region A2, corresponding to the FeP phase. (**f**) HAADF-STEM image and (**g**–**i**) corresponding EDS-based elemental mapping images of CFP NPs.

TEM investigation indicated considerable morphological changes after the phosphorization procedure. Initially, the CFO NPs have a clearly defined nanosheet-like shape (Figure 4a). However, after phosphorization, the nanosheet structure is severely damaged, resulting in an uneven and fragmented shape (Figure 4b). High-resolution TEM (HRTEM) reveals additional structural changes, demonstrating that the CFP NPs contain both crystalline and amorphous areas (Figure 4c). The crystalline portions show distinct lattice fringes with interplanar spacings of 0.189 nm, corresponding to the (211) plane of CoP, and 0.24 nm, matching the (111) plane of FeP (Figure 4d,e) [37,43]. These findings demonstrate the presence of CoP and FeP phases in CFP NPs. EDS elemental mapping (Figure 4f–i and Figure S4) confirms these findings by revealing a homogeneous distribution of Fe, Co, and P throughout the CFP NPs. This constant dispersion of elements throughout the particles suggests that CFO NPs were successfully converted into CFP NPs via the phosphorization process, which preserved the material’s elemental composition while dramatically altering its shape and crystalline structure.

### 3.2. Electrochemical OER Performance Evaluation

**Figure 5:**
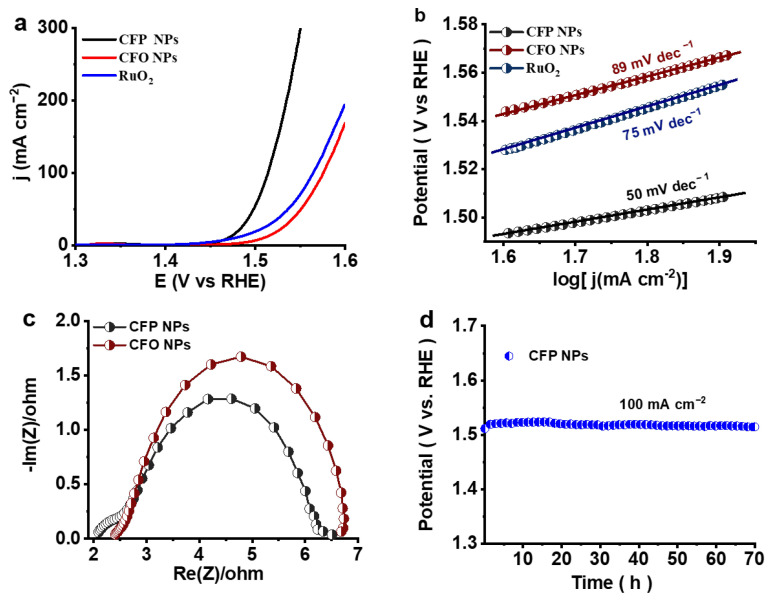
(**a**) The OER polarization curve of CFP NPs, CFO NPs, and RuO_2_ and (**b**) corresponding Tafel slopes. (**c**) Electrochemical impedance spectroscopy (EIS). (**d**) Long-term stability test performed at a constant current density of 100 mA cm^−2^.

Using linear sweep voltammetry (LSV) in a conventional three-electrode system with an oxygen-saturated 1 M KOH electrolyte, the electrocatalytic activity of CFP NPs was assessed. The OER activity of the synthesized catalysts (CFP, CFO, and commercial RuO_2_) was evaluated in a 1 M KOH solution using NF as the substrate. The LSV curves (Figure 5a) reveal that CFP NPs exhibit superior OER performance compared to CFO NPs and commercial RuO_2_. Specifically, the CFP NP catalyst demonstrates a significantly lower overpotential, indicating enhanced catalytic activity. At a current density of 100 mA cm^−2^, the CFP NP catalyst requires an overpotential of only 284 mV, outperforming both commercial RuO_2_ (335 mV) and CFO NPs (345 mV). The resulting nanostructured CFP NP catalyst exhibits high OER activity due to its increased electrochemical surface area and abundance of exposed active sites [45]. During the OER process, Fe and Co serve as active centers. A Co electron donation to Fe optimizes the electronic structure, enhancing the interaction with oxygen intermediates, which in turn improves the overall catalytic performance [46,47]. Additionally, phosphorus in the CFP NP structure plays a crucial role by drawing electrons away from the metal surface and increasing the positive charge on the Co and Fe active sites [45]. This electron withdrawal enhances the adsorption of protons and oxygen-containing intermediates, thereby accelerating the reaction kinetics [48].

In addition, the unique amorphous/crystalline composite structure of the CFP NPs is responsible for their remarkable OER performance. There are many active sites for the OER due to the high density of defects and unsaturated bonds produced by the amorphous phase, which is distinguished by a large specific surface area and long-range disordered structure. The catalyst and reactants can interact more effectively thanks to these structural characteristics, which greatly increase the catalytic activity. Furthermore, because of their outstanding electrical conductivity and deliberate growth on the amorphous phase, the crystalline particles ensure enhanced reaction kinetics and quick electron transfer. Together with improving the overall catalytic OER performance, this synergistic interaction between the crystalline and amorphous phases also increases the catalyst’s longevity under these operating circumstances. The superior electrocatalytic behavior of the CFP NPs is confirmed by the outcomes of successive Tafel tests, adding credence to the veracity of this explanation (Figure 5b). The CFP NP catalyst exhibits a Tafel slope of 50 mV dec^−1^, indicating rapid OER kinetics. In comparison, commercial RuO_2_ and CFO NPs show higher Tafel slopes of 75 mV dec^−1^ and 89 mV dec^−1^, respectively, underscoring the superior catalytic efficiency of the CFP NPs. The distinct amorphous/crystalline structure of CFP NPs greatly improves their electrocatalytic capabilities, as evidenced by the decreased Tafel slope, which also suggests a faster reaction rate and greater performance in assisting the OER. This enhancement is ascribed to the effective charge transfer made possible by the structural characteristics of CFP NPs, as well as the improved interaction between the catalyst and the reactants. The charge transfer properties of the catalysts were evaluated using electrochemical impedance spectroscopy (EIS) measurements (Figure 5c). The CFP NPs had a significantly lower charge-transfer resistance than CFO NPs, according to the EIS spectra. This indicates improved electronic conductivity and more effective charge transfer kinetics at the electrode–electrolyte interface during the OER. The improved ability of CFP NPs to assist in electron transfer and speed up the rate of the reaction is demonstrated by their decreased charge-transfer resistance. This is further supported by the fact that the CFP NPs’ smaller semicircle in the Nyquist plot shows less resistance to charge transfer, implying that the NPs’ structural and electrical characteristics influence their efficient catalytic activity. This increased charge transfer is critical for maximizing the electrocatalytic activity and efficiency of the CFP NPs in the OER process.

The CFP catalyst was tested for stability and endurance using chronopotentiometry at a constant current density of 100 mA cm^−2^, in addition to its high OER activity. The CFP NPs were exceptionally stable, retaining steady performance with no appreciable degradation seen after 70 h of continuous operation (Figure 5d). This outstanding longevity demonstrates the catalyst’s suitability for prolonged usage in the oxygen evolution processes. The CFP NPs exhibited minimal performance loss and maintained a consistent voltage output throughout the test, indicating their capacity for long-term use without significant degradation. This robustness emphasizes their appropriateness for practical applications requiring long-term stability and performance, making them highly promising materials for sustainable and efficient electrocatalytic OER processes.

Following the long-term electrocatalysis stability testing, XPS analysis provided further insight into the chemical composition of the post-OER samples (Figure S5). These findings confirmed the formation of cobalt–iron oxides/oxyhydroxides during the OER process in an alkaline environment. The post-OER samples exhibited a significant blue shift in the Co and Fe XPS spectra, indicating that the valence states of the catalysts were higher than those of the as-prepared CFP NPs during the OER (Figure S5a,b). Additionally, XPS analysis confirmed the disappearance of low-energy peaks at 129.03 eV and 129.78 eV, corresponding to the P 2p_3/2_ and P 2p_1/2_ states in the CFP NPs (Figure S5c). This indicates that the surface oxidation of CoFeP occurred, leading to the formation of cobalt–iron oxides/oxyhydroxides during the OER process [49]. Initially, the XPS spectrum for phosphorus showed peaks at 133.3 eV and 134.1 eV, both attributable to P–O bonds. After the stability tests, the peak at 133.3 eV disappeared completely, while the peak at 134.1 eV decreased significantly. This suggests that the CFP NPs were not fully oxidized to cobalt–iron oxides/oxyhydroxides even after 70 h of OER testing, with a small amount of phosphorus oxides likely remaining.

These findings indicate that metal phosphide- and phosphate-based electrocatalysts are unstable under practical alkaline OER conditions, as they often degrade into metal oxides/oxyhydroxides due to phosphorus leaching [50]. This leaching leads to structural reorganization, increased surface area, and roughening as smaller oxide/oxyhydroxide anions replace the larger phosphorus anions [50,51]. Despite this transformation, the CFP NPs catalyst outperformed native metal oxides/oxyhydroxides (CFO NPs) in long-term stability testing, likely due to its enhanced surface roughness and increased number of active sites.

## 4. Conclusions

The development of non-precious metal electrocatalysts is critical for increasing the efficiency and cost-effectiveness of renewable energy sources, notably in water-splitting systems. This study describes a simple and cost-effective method for converting cobalt–iron oxide/hydroxide (CFO NPs) into CoFeP nanoparticles (CFP NPs) via phosphorization. The generated CFP NPs exhibit outstanding electrocatalytic efficiency with a low overpotential of 284 mV at a current density of 100 mA cm^−2^, greatly surpassing both the CFO NP precursor and the commercial RuO_2_ catalyst. The CFP NPs show outstanding catalytic activity due to their unique amorphous/crystalline composite structure, which improves both electronic conductivity and active site density. The inclusion of cobalt and iron promotes electronic interactions, whereas phosphorus improves reactant adsorption, resulting in enhanced OER performance. Post-OER characterization revealed transformations, including the formation of cobalt–iron oxides/oxyhydroxides. Despite these changes, the CFP NPs demonstrated exceptional long-term stability, maintaining a consistent performance over 70 h of continuous operation. This stability, coupled with their enhanced surface roughness and active site availability, underscores the practical applicability of CFP NPs for prolonged use. This study not only demonstrates the potential of CFP NPs as an efficient and long-lasting electrocatalyst for OER, but it also presents a diverse synthetic technique for creating improved bimetallic phosphide catalysts. These discoveries pave the way for future advances in electrocatalysis and sustainable energy technologies, thereby facilitating the shift to more cost-effective and environmentally friendly hydrogen generation techniques.

## Data Availability

All the experimental data presented within this article along with the Supplementary Materials will be made available from the authors upon reasonable request.

## Supplementary Materials

The following are available online at https://www.mdpi.com/article/10.3390/nano14171384/s1, Figure S1: Calibration of the Hg/HgO reference electrode; Figure S2: SEM image, atomic and weight percentage acquired by EDS of CFO NPs. (a) SEM image of the regions and (b) EDS results acquired from the area; Figure S3: SEM image and atomic and weight percentage acquired by EDS of CFP NPs. (a) SEM image of the regions and (b) EDS results acquired from the area; Figure S4: TEM image and atomic and weight percentage acquired by EDS of CFP NPs. (a) TEM image of the regions and (b) EDS results acquired from the area; and Figure S5: XPS spectra of the as-prepared CFP NP catalyst subjected to OER electrolysis at a constant current density of 100 mA cm^−2^ for 70 h. (a) Co 2p, (b) Fe 2p, (c) P 2p, and (d) O 1s.

## References

[1] Saeedmanesh A., Mac Kinnon M.A., Brouwer J. (2018). Hydrogen is essential for sustainability. Curr. Opin. Electrochem..

[2] Ozcan H., El-Emam R.S., Celik S., Amini Horri B. (2023). Recent advances, challenges, and prospects of electrochemical water-splitting technologies for net-zero transition. Clean. Chem. Eng..

[3] Mehtab A., Ali S.A., Sadiq I., Shaheen S., Khan H., Fazil M., Pandit N.A., Naaz F., Ahmad T. (2024). Hydrogen Energy as Sustainable Energy Resource for Carbon-Neutrality Realization. ACS Sustain. Resour. Manag..

[4] Raveendran A., Chandran M., Dhanusuraman R. (2023). A comprehensive review on the electrochemical parameters and recent material development of electrochemical water splitting electrocatalysts. RSC Adv..

[5] Xu X., Zhong Y., Wajrak M., Bhatelia T., Jiang S.P., Shao Z. (2024). Grain boundary engineering: An emerging pathway toward efficient electrocatalysis. InfoMat.

[6] Wang S., Lu A., Zhong C.-J. (2021). Hydrogen production from water electrolysis: Role of catalysts. Nano Converg..

[7] Zhu J., Hu L., Zhao P., Lee L.Y.S., Wong K.-Y. (2020). Recent Advances in Electrocatalytic Hydrogen Evolution Using Nanoparticles. Chem. Rev..

[8] Chen Z., Wei W., Ni B.-J. (2021). Cost-effective catalysts for renewable hydrogen production via electrochemical water splitting: Recent advances. Curr. Opin. Green Sustain. Chem..

[9] Salonen L.M., Petrovykh D.Y., Kolen’ko Y.V. (2021). Sustainable catalysts for water electrolysis: Selected strategies for reduction and replacement of platinum-group metals. Mater. Today Sustain..

[10] Fan H., Chen W., Chen G., Huang J., Song C., Du Y., Li C., Ostrikov K. (2020). Plasma-heteroatom-doped Ni-V-Fe trimetallic phospho-nitride as high-performance bifunctional electrocatalyst. Appl. Catal. B.

[11] Li J., Zou S., Liu X., Lu Y., Dong D. (2020). Electronically Modulated CoP by Ce Doping as a Highly Efficient Electrocatalyst for Water Splitting. ACS Sustain. Chem. Eng..

[12] Deng Y., Cao Y., Xia Y., Xi X., Wang Y., Jiang W., Yang D., Dong A., Li T. (2022). Self-Templated Synthesis of CoFeP@C Cage-In-Cage Superlattices for Enhanced Electrocatalytic Water Splitting. Adv. Energy Mater..

[13] Guo M., Yuan Y., Qu Y., Yu T., Yuan C., Lu Z.-h. (2022). Porous N-doped carbon with confined Fe-doped CoP grown on CNTs for superefficient oxygen evolution electrocatalysis. Chem. Commun..

[14] Gao J., Li Y., Yu X., Ma Y. (2022). Graphdiyne reinforced multifunctional Cu/Ni bimetallic Phosphides-Graphdiyne hybrid nanostructure as high performance electrocatalyst for water splitting. J. Colloid Interface Sci..

[15] Meena A., Thangavel P., Jeong D.S., Singh A.N., Jana A., Im H., Nguyen D.A., Kim K.S. (2022). Crystalline-amorphous interface of mesoporous Ni_2_P@FePO_x_H_y_ for oxygen evolution at high current density in alkaline-anion-exchange-membrane water-electrolyzer. Appl. Catal. B.

[16] Xiong D., Lu C., Chen C., Wang J., Kong Y., Liu T., Ying S., Yi F.-Y. (2022). CoFeP nanocube-arrays based on Prussian blue analogues for accelerated oxygen evolution electrocatalysis. J. Power Sources.

[17] Wang Y., Wang S., Chen X., Zhao X., Chang S., Guo F., Xu J., Shang Y., Zhang Y. (2021). An etch-doping strategy: Cobalt–iron bimetallic phosphide as a bifunctional electrocatalyst for highly efficient water splitting. New J. Chem..

[18] He B., Deng Q., Wang Y.-C., Tang Y.-W., Hao Q.-L., Liu H.-K., Su Z. (2022). Modification of surface electronic structure via Ru-doping: Porous Ru–CoFeP nanocubes to boost the oxygen evolution reaction. J. Power Sources.

[19] Meena A., Shin G., Cho S., Singh A.N., Aqueel Ahmed A.T., Jana A., Kim H., Im H. (2023). Engineering a synergistic CoMn-LDH/Fe_2_O_3_@NF heterostructure for highly efficient oxygen evolution reaction. Ceram. Int..

[20] Ahmed A.T., Ansari A.S., Sree V.G., Jana A., Meena A., Sekar S., Cho S., Kim H., Im H. (2023). Nitrogen-Doped CuO@CuS Core–Shell Structure for Highly Efficient Catalytic OER Application. Nanomaterials.

[21] Dutta A., Pradhan N. (2017). Developments of Metal Phosphides as Efficient OER Precatalysts. J. Phys. Chem..

[22] Trotochaud L., Young S.L., Ranney J.K., Boettcher S.W. (2014). Nickel–Iron Oxyhydroxide Oxygen-Evolution Electrocatalysts: The Role of Intentional and Incidental Iron Incorporation. J. Am. Chem. Soc..

[23] Li X., Hao X., Abudula A., Guan G. (2016). Nanostructured catalysts for electrochemical water splitting: Current state and prospects. J. Mater. Chem. A.

[24] Singh A.N., Hajibabaei A., Ha M., Meena A., Kim H.-S., Bathula C., Nam K.-W. (2023). Reduced Potential Barrier of Sodium-Substituted Disordered Rocksalt Cathode for Oxygen Evolution Electrocatalysts. Nanomaterials.

[25] Han X., Yu C., Yang J., Zhao C., Huang H., Liu Z., Ajayan P.M., Qiu J. (2016). Mass and Charge Transfer Coenhanced Oxygen Evolution Behaviors in CoFe-Layered Double Hydroxide Assembled on Graphene. Adv. Mater. Interfaces.

[26] Abdelghafar F., Xu X., Guan D., Lin Z., Hu Z., Ni M., Huang H., Bhatelia T., Jiang S.P., Shao Z. (2024). New Nanocomposites Derived from Cation-Nonstoichiometric Ba_x_(Co, Fe, Zr, Y)O_3−δ_ as Efficient Electrocatalysts for Water Oxidation in Alkaline Solution. ACS Mater. Lett..

[27] Zou T., Wang Y., Xu F. (2023). Defect-Engineered Charge Transfer in a PtCu/Pr_x_Ce_1–x_O_2_ Carbon-Free Catalyst for Promoting the Methanol Oxidation and Oxygen Reduction Reactions. ACS Appl. Mater. Interfaces.

[28] Liu X., Huang C., Ouyang B., Du Y., Fu B., Du Z., Ju Q., Ma J., Li A., Kan E. (2022). Enhancement of Mass and Charge Transfer during Carbon Dioxide Photoreduction by Enhanced Surface Hydrophobicity without a Barrier Layer. Chem. Eur. J..

[29] Wang X., Yu X., Wu S., He P., Qin F., Yao Y., Bai J., Yuan G., Ren L. (2023). Crystalline–Amorphous Interface Coupling of Ni_3_S_2_/NiP_x_/NF with Enhanced Activity and Stability for Electrocatalytic Oxygen Evolution. ACS Appl. Mater. Interfaces.

[30] Tahir M., Pan L., Idrees F., Zhang X., Wang L., Zou J.-J., Wang Z.L. (2017). Electrocatalytic oxygen evolution reaction for energy conversion and storage: A comprehensive review. Nano Energy.

[31] Wan Y., Wang Z., Zheng M., Li J., Lv R. (2023). Heterogeneous crystalline–amorphous interface for boosted electrocatalytic nitrogen reduction to ammonia. J. Mater. Chem. A.

[32] Jourshabani M., Lee B.-K. (2021). Unmasking the Role of an Amorphous/Amorphous Interface and a Crystalline/Amorphous Interface in the Transition of Charge Carriers on the CN/SiO_2_/WO_3_ Photocatalyst. ACS Appl. Mater. Interfaces.

[33] Bathula C., Meena A., Sekar S., Singh A.N., Soni R., El-Marghany A., Palem R.R., Kim H.-S. (2023). Self-Assembly of Copper Oxide Interfaced MnO_2_ for Oxygen Evolution Reaction. Nanomaterials.

[34] Zhao X., Guo X., Yang Z., Liu H., Qian Q. (2011). Phase-controlled preparation of iron (oxyhydr)oxide nanocrystallines for heavy metal removal. J. Nanopart. Res..

[35] Si Y., Guo C., Xie C., Xiong Z. (2018). An Ultrasonication-Assisted Cobalt Hydroxide Composite with Enhanced Electrocatalytic Activity toward Oxygen Evolution Reaction. Materials.

[36] Huo W., Li L., Zhang Y., Li J., Xu Q., Zhang B., Zhang L., Li X. (2019). Monodispersed Hierarchical γ-AlOOH/Fe(OH)_3_ Micro/Nanoflowers for Efficient Oxygen Evolution Reaction. Front. Mater..

[37] Fu R., Jiao Q., Yang C., Jiao X., Zhang X., Feng C., Li H., Zhang Y., Zhao Y. (2023). CoFeP/NC@CoP/Ni_2_P heterostructure for efficient overall water splitting. New J. Chem..

[38] Cai X., Song Q., Jiao D., Yu H., Tan X., Wang R., Luo S. (2022). Bifunctional electrocatalysts of CoFeP/rGO heterostructure for water splitting. Int. J. Hydrogen Energy.

[39] Zhao R., Ni B., Wu L., Sun P., Chen T. (2022). Carbon-based iron-cobalt phosphate FeCoP/C as an effective ORR/OER/HER trifunctional electrocatalyst. Colloids Surf. A Physicochem. Eng. Asp..

[40] Zhan W., Ma L., Gan M., Xie F. (2022). Ultra-fine bimetallic FeCoP supported by N-doped MWCNTs Pt-based catalyst for efficient electrooxidation of methanol. Appl. Surf. Sci..

[41] Xiang R., Duan Y., Tong C., Peng L., Wang J., Shah S.S.A., Najam T., Huang X., Wei Z. (2019). Self-standing FeCo Prussian blue analogue derived FeCo/C and FeCoP/C nanosheet arrays for cost-effective electrocatalytic water splitting. Electrochim. Acta..

[42] Yan Y., Lin J., Bao K., Xu T., Qi J., Cao J., Zhong Z., Fei W., Feng J. (2019). Free-standing porous Ni2P-Ni5P4 heterostructured arrays for efficient electrocatalytic water splitting. J. Colloid Interface Sci..

[43] Yang F., Yang S., Wang X., Li Z., Song Z., Ma Y., Liu C., Song L. (2022). Fabrication of robust CoP/Ni2P/CC composite for efficient hydrogen evolution reaction and the reduction of graphene oxide. Int. J. Hydrogen Energy.

[44] Li D., Zhou C., Yang R., Xing Y., Xu S., Jiang D., Tian D., Shi W. (2021). Interfacial Engineering of the Co_x_P–Fe_2_P Heterostructure for Efficient and Robust Electrochemical Overall Water Splitting. ACS Sustain. Chem. Eng..

[45] Zhang Y., Gao T., Jin Z., Chen X., Xiao D. (2016). A robust water oxidation electrocatalyst from amorphous cobalt–iron bimetallic phytate nanostructures. J. Mater. Chem. A.

[46] Wen L., Zhang X., Liu J., Li X., Xing C., Lyu X., Cai W., Wang W., Li Y. (2019). Cr-Dopant Induced Breaking of Scaling Relations in CoFe Layered Double Hydroxides for Improvement of Oxygen Evolution Reaction. Small.

[47] Zhang T., Jiang J., Sun W., Gong S., Liu X., Tian Y., Wang D. (2024). Spatial configuration of Fe–Co dual-sites boosting catalytic intermediates coupling toward oxygen evolution reaction. Proc. Natl. Acad. Sci. USA.

[48] Enkhtuvshin E., Kim K.M., Kim Y.-K., Mihn S., Kim S.J., Jung S.Y., Thu Thao N.T., Ali G., Akbar M., Chung K.Y. (2021). Stabilizing oxygen intermediates on redox-flexible active sites in multimetallic Ni–Fe–Al–Co layered double hydroxide anodes for excellent alkaline and seawater electrolysis. J. Mater. Chem. A.

[49] Xu J., Li J., Xiong D., Zhang B., Liu Y., Wu K.-H., Amorim I., Li W., Liu L. (2018). Trends in activity for the oxygen evolution reaction on transition metal (M = Fe, Co, Ni) phosphide pre-catalysts. Chem. Sci..

[50] Wygant B.R., Kawashima K., Buddie Mullins C. (2018). Catalyst or Precatalyst? The Effect of Oxidation on Transition Metal Carbide, Pnictide, and Chalcogenide Oxygen Evolution Catalysts. ACS Energy Lett..

[51] Chen W., Wang H., Li Y., Liu Y., Sun J., Lee S., Lee J.-S., Cui Y. (2015). In Situ Electrochemical Oxidation Tuning of Transition Metal Disulfides to Oxides for Enhanced Water Oxidation. ACS Cent. Sci..

