# Supplementary material for: Highly Efficient CoFeP Nanoparticle Catalysts for Superior Oxygen Evolution Reaction Performance
Source: Nanomaterials (Basel). 2024 Aug 24;14(17):1384. doi: 10.3390/nano14171384 (PMC11396991; doi:10.3390/nano14171384)
Supplement: Supplementary file 1 [file nanomaterials-14-01384-s001.zip › nanomaterials-3179373-supplementary.pdf]

nanomaterials

Supplementary Materials

Highly Efficient CoFeP Nanoparticle Catalysts for Superior Oxygen Evolution Reaction Performance

Abhishek Meena [1], Abu Talha Aqueel Ahmed [1], Aditya Narayan Singh [2], Vijaya Gopalan Sree [3], Hyunsik Im [1] and Sangeun Cho [1,*]

[1] Division of System Semiconductor, College of AI Convergence, Dongguk University, Seoul 04620, Republic of Korea

[2] Department of Energy and Materials Engineering, Dongguk University, Seoul 04620, Republic of Korea

[3] Department of Physics, Dongguk University, Seoul 04620, Republic of Korea

* **Correspondence:** sangeun.c@dongguk.edu

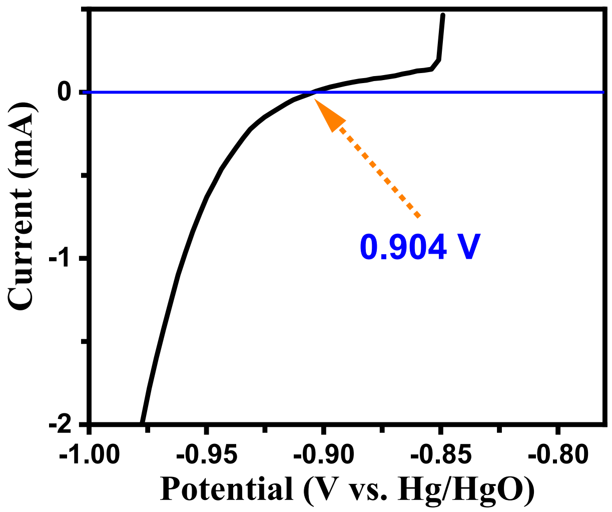

Figure S1. Calibration of the Hg/HgO reference electrode. The calibration of the Hg/HgO reference electrode involved conducting measurements using a high-purity H_2-saturated electrolyte with a Pt wire as the working electrode. Linear sweep voltammetry (LSV) was performed at a scan rate of 1 mV/s, and the potential at zero current was considered the thermodynamic potential for the hydrogen evolution reaction (HER). Consequently, in a 1 M KOH solution, the relationship between the RHE and Hg/HgO electrode is given by E(RHE) = E(Hg/HgO) + 0.904.

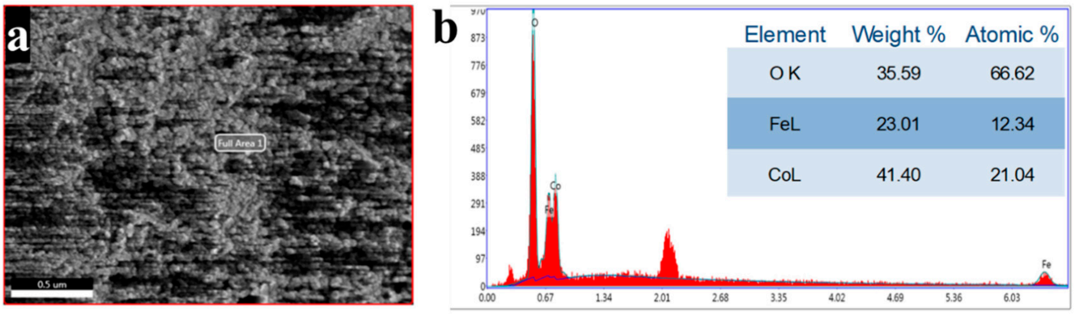

Figure S2. SEM image, atomic and weight percentage acquired by EDS of CFO NPs. **(a)** SEM image of the regions and **(b)** EDS results acquired from the area.

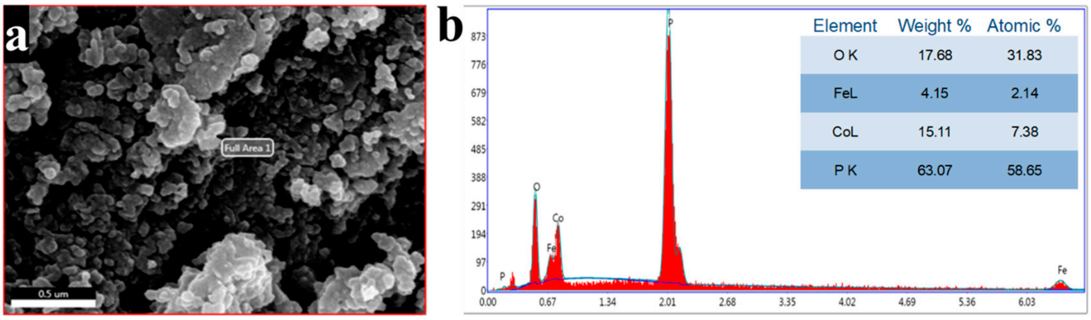

Figure S3. SEM image and atomic and weight percentage acquired by EDS of CFP NPs. **(a)** SEM image of the regions and **(b)** EDS results acquired from the area.

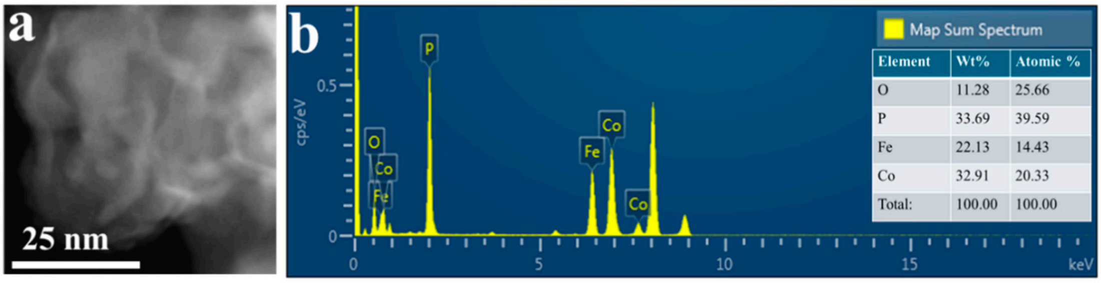

Figure S4. TEM image and atomic and weight percentage acquired by EDS of CFP NPs. **(a)** TEM image of the regions and **(b)** EDS results acquired from the area.

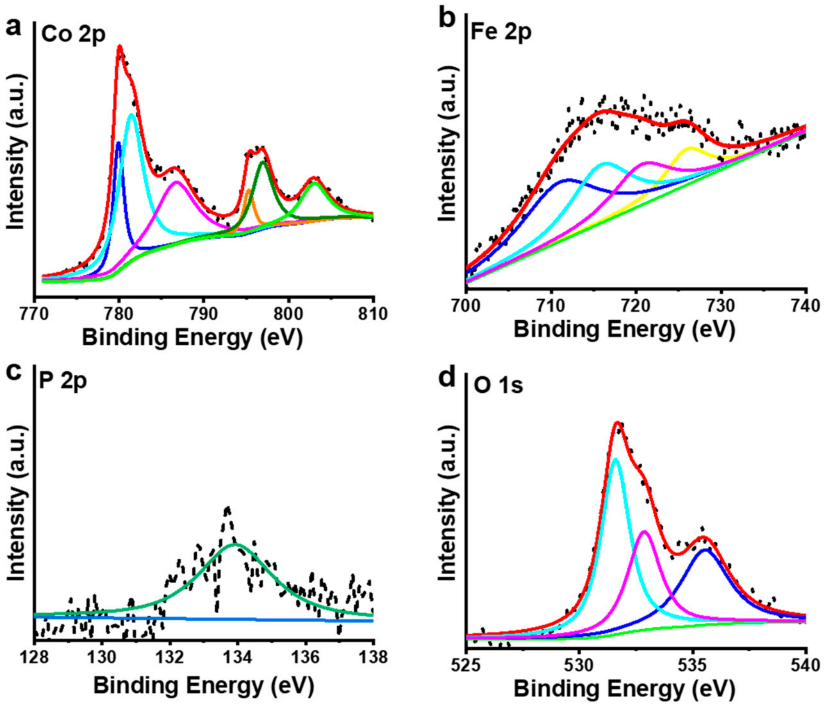

Figure S5. XPS spectra of the as-prepared CFP NPs catalyst subjected to OER electrolysis at a constant current density of 100 mA cm^{-2} for 70 h. **(a)** Co 2p, **(b)** Fe 2p, **(c)** P 2p and **(d)** O 1s.
